# Mapping the knowledge structure and emerging trends of blood-based biomarkers in autism spectrum disorder: A bibliometric analysis (2016–2026)

**DOI:** 10.1016/j.ibneur.2026.09.012

**Published:** 2026-09-24

**Authors:** Lei Xu, Jia-Ying Zhang, Hong-Biao Huang, Yuan-Yuan Huang, Yi-Yuan Chen, Ying Wu, Ran Wei, Hui Zhong, Bo-Qiu Yan, Hai-Tao Lv, Hua Jin

**Affiliations:** a Department of Child Health Care, Suzhou Municipal Hospital, The Affiliated Suzhou Hospital of Nanjing Medical University, Suzhou, China; b Department of Cardiology, Children’s Hospital of Soochow University, Suzhou, China; c Department of Pediatrics, Fujian Provincial Hospital, Fuzhou University Affiliated Provincial Hospital, Fuzhou, China; d Department of Child Health Care, Children’s Hospital of Soochow University, Suzhou, China

**Keywords:** Autism spectrum disorder, Blood-based biomarkers, Bibliometric analysis, Mitochondrial dysfunction, Gut microbiota

## Abstract

**Background:**

Autism spectrum disorder (ASD) is a clinically heterogeneous neurodevelopmental condition, and its diagnosis still relies mainly on behavioral assessment. Blood-based biomarkers may provide objective support for early detection, biological subgrouping, clinical monitoring, and individualized intervention. However, findings in this field remain dispersed across multiple biological pathways. This study aimed to map the knowledge structure and emerging trends of ASD blood-based biomarker research.

**Methods:**

Publications on ASD blood-based biomarkers from 2016 to 2026 were retrieved from the Web of Science Core Collection. After duplicate removal and eligibility screening, 1785 English-language articles and reviews were included. CiteSpace, VOSviewer, and Microsoft Excel were used to examine publication trends, collaboration networks, influential journals and authors, co-cited references, keyword clusters, and citation bursts.

**Results:**

Research output increased steadily, with the United States and China contributing the largest number of publications and the United States showing stronger international connectivity. Co-cited reference and keyword analyses identified oxidative stress, inflammatory cytokines, gut microbiota, metabolomics, mitochondrial respiration, and gastrointestinal symptoms as major knowledge clusters. Recent burst terms, including machine learning, Mendelian randomization, gut–brain axis, innate immunity, sex differences, and neurological disorders, indicate a shift toward causal inference, multi-omics integration, and data-driven biomarker prediction.

**Conclusions:**

ASD blood-based biomarker research has grown steadily over the past decade and is moving from descriptive clinical observations toward mechanistic, multi-omics, and computational approaches. Oxidative stress, mitochondrial dysfunction, gut–brain interactions, inflammatory responses, and machine learning-based prediction now represent major directions in this field. Future studies should focus on multicenter longitudinal cohorts, standardized analytical workflows, detailed clinical phenotyping, and independent validation to improve reproducibility and support clinical translation.

## Highlights

• Research on blood-based biomarkers in autism has grown over the past decade.
• Oxidative stress and mitochondrial dysfunction are long-standing research topics.
• Gut microbiota and metabolomics have received growing research attention.
• Machine learning has become a recent focus in biomarker research.
• Multicenter validation is essential for moving biomarkers toward clinical use.

## Introduction

Autism spectrum disorder (ASD) is a complex neurodevelopmental condition that usually emerges in early childhood and may persist throughout life, affecting social communication, adaptive behavior, and daily functioning (Baio et al., 2018, Lord et al., 2020). With its rising global prevalence, ASD has become an important public health concern (Tafolla et al., 2025). At present, clinical diagnosis still relies largely on behavioral assessments and standardized scales, which may be influenced by age, developmental level, symptom heterogeneity, and evaluator experience. This diagnostic reality has increased the need for objective biological indicators that can complement clinical evaluation.

Among potential biomarker sources, blood-based biomarkers are particularly relevant because they are relatively accessible and suitable for large-scale clinical studies. These indicators may help support early detection, biological subgrouping, clinical monitoring, and future individualized intervention strategies. In recent years, advances in multi-omics technologies have further accelerated biomarker discovery in ASD, drawing increasing attention to peripheral biological indicators, including blood-based molecular, metabolic, inflammatory, and microbiota-related markers (Ecker et al., 2015, Huang et al., 2026, Ristori et al., 2020). Despite the rapid expansion of ASD biomarker research, findings remain heterogeneous across studies. The growing number of publications has made it increasingly difficult to obtain a clear overview of the field, especially in terms of publication trends, collaboration patterns, knowledge structure, and emerging research priorities. Existing reviews have mainly discussed biological mechanisms, while quantitative analyses focusing specifically on ASD blood-based biomarkers remain limited.

Bibliometric analysis offers a useful approach for tracing research development, mapping collaboration networks, and identifying the evolution of knowledge within a specific field (Chen, 2006, Donthu et al., 2021). Previous bibliometric studies on ASD have mainly focused on neuroimaging, genetics, gut microbiota, or inflammation-related topics, leaving the literature on blood-based biomarkers less systematically characterized (Chen et al., 2025, Liu et al., 2026, Lu et al., 2022). Given the increasing attention to this topic, a systematic bibliometric analysis may provide a clearer understanding of current research hotspots and emerging trends.

Against this background, the present study used bibliometric and visual analyses to examine publications on ASD blood-based biomarkers from 2016 to 2026. By mapping publication trends, collaboration networks, co-cited references, and keyword evolution, this study sought to clarify the knowledge structure of the field and identify research directions that are likely to shape future biomarker studies.

## Methods

A literature search was conducted in the Web of Science Core Collection (WoSCC) database to identify studies on ASD and blood-based biomarkers. The search strategy was developed with reference to previous bibliometric studies and refined through repeated testing. The following formula was used: TS = (("autism spectrum disorder*" OR ASD OR autism OR autistic OR "autistic disorder*" OR "pervasive developmental disorder*") AND (biomarker* OR "biological marker*" OR "diagnostic marker*" OR "molecular marker*" OR "blood-based biomarker*" OR "circulating biomarker*" OR proteomic* OR metabolomic* OR transcriptomic* OR cytokine* OR chemokine* OR metabolite* OR protein* OR miRNA OR microRNA OR "oxidative stress" OR inflammation OR inflammatory) AND (blood OR serum OR plasma OR "whole blood" OR "peripheral blood" OR circulating)).

The search covered publications from January 1, 2016, to May 19, 2026. Duplicate records were removed first (n = 0). The remaining records were screened by document type and language. Only English-language Articles and Review Articles were included. Editorial Materials, Meeting Abstracts, Early Access and Letters were excluded (n = 7). Two researchers independently screened the titles and abstracts under the supervision of the corresponding author. Disagreements were resolved through discussion, and a third researcher was consulted when consensus was not reached.

All searches and data exports were completed on May 19, 2026. Records were exported in plain text format with “Full Record and Cited References” selected. A total of 1785 publications met the eligibility criteria and were included in the final bibliometric analysis (Fig. 1).

**Fig. 1:**
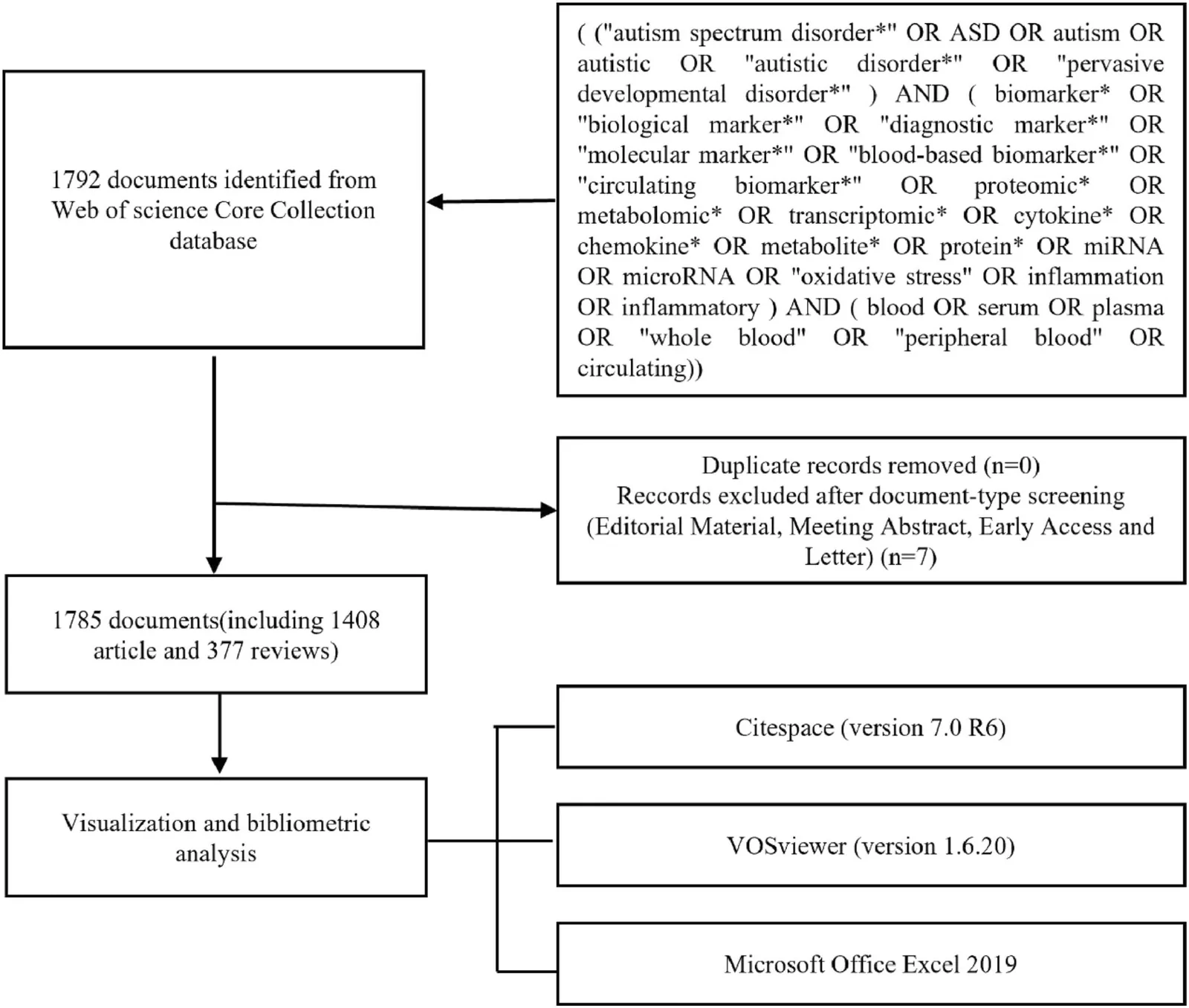
Flowchart of literature screening and selection.

The extracted data included publication year, authors, countries/regions, institutions, journals, citations, references, and keywords. CiteSpace (version 7.0.R6) and VOSviewer (version 1.6.20) were used for bibliometric analysis and visualization. CiteSpace was used to analyze country/region and institutional collaboration networks, journal co-citation patterns, co-cited references, reference and keyword clustering, timeline visualization, and citation/keyword bursts. The search was not restricted to human studies and included both clinical human and preclinical animal-model research. For the stratified analysis, review articles were excluded, and keyword clustering was performed separately for the two groups. VOSviewer was used to construct the author collaboration network and co-cited author network. Microsoft Office Excel 2019 was used to summarize annual publication trends and organize basic bibliometric data.

After screening according to the predefined eligibility criteria, 1785 publications were included in the final bibliometric analysis.

## Results

### Annual publication trends

The final dataset comprised 1785 publications, including 1408 original articles (78.9%) and 377 reviews (21.1%). Research output showed an overall upward trend during the study period, despite some year-to-year variation. Annual publications increased from 103 in 2016 to 238 in 2025. Data for 2026 were incomplete because the literature search was conducted through May 19, 2026, and were therefore not directly comparable with those of previous full years (Fig. 2).

**Fig. 2:**
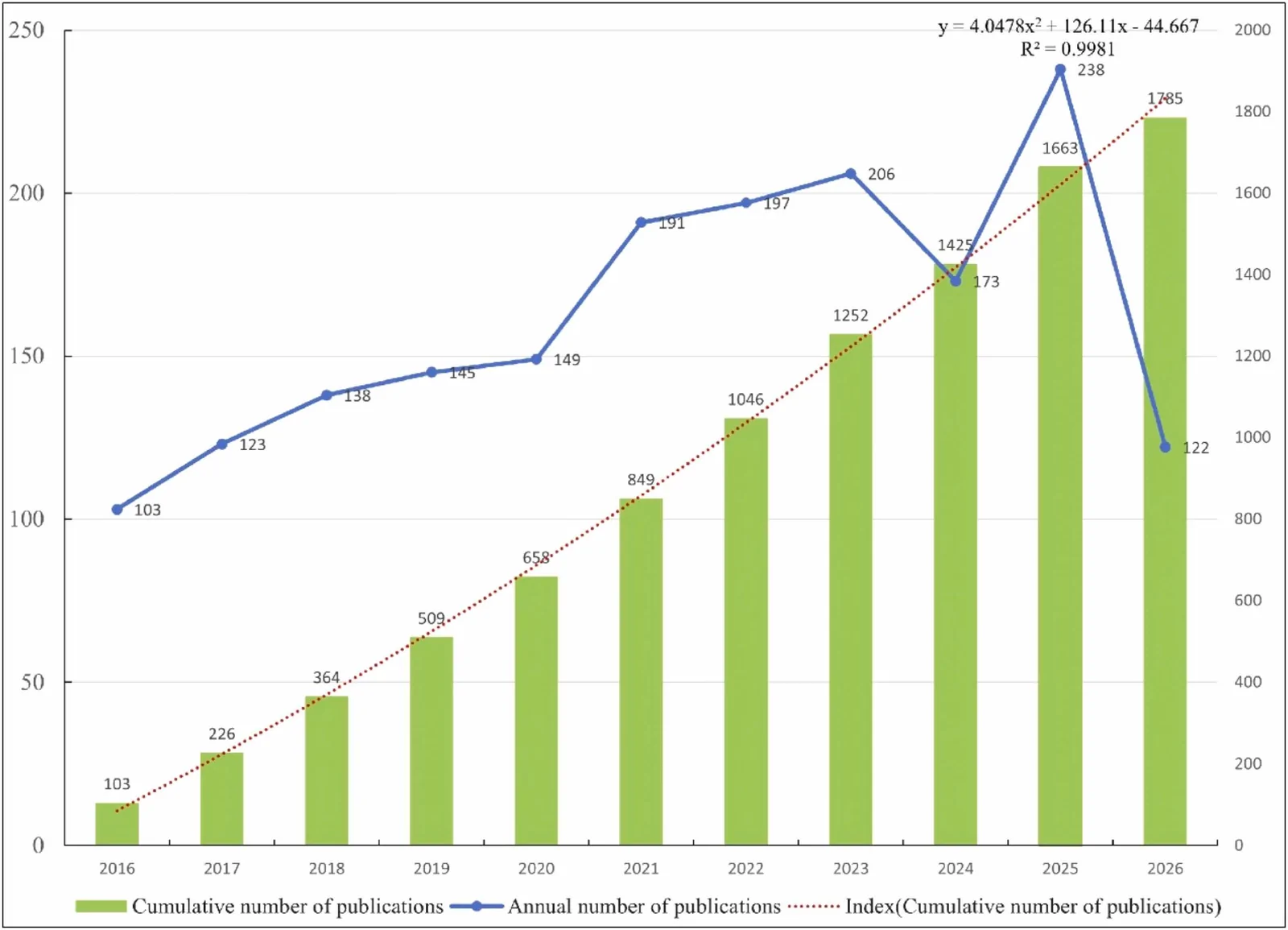
Annual publication trends.

A total of 1785 publications were identified during the study period. Both annual and cumulative publication outputs showed an overall upward trend.

### Analysis of countries/regions

Research on ASD and blood-based biomarkers involved contributions from 96 countries and regions. The collaboration network generated by CiteSpace is presented in Fig. 3, in which each node represents a country or region, node size reflects publication output, and connecting lines indicate collaborative relationships between countries/regions. The United States and China were identified as the leading contributors in terms of publication volume.

**Fig. 3:**
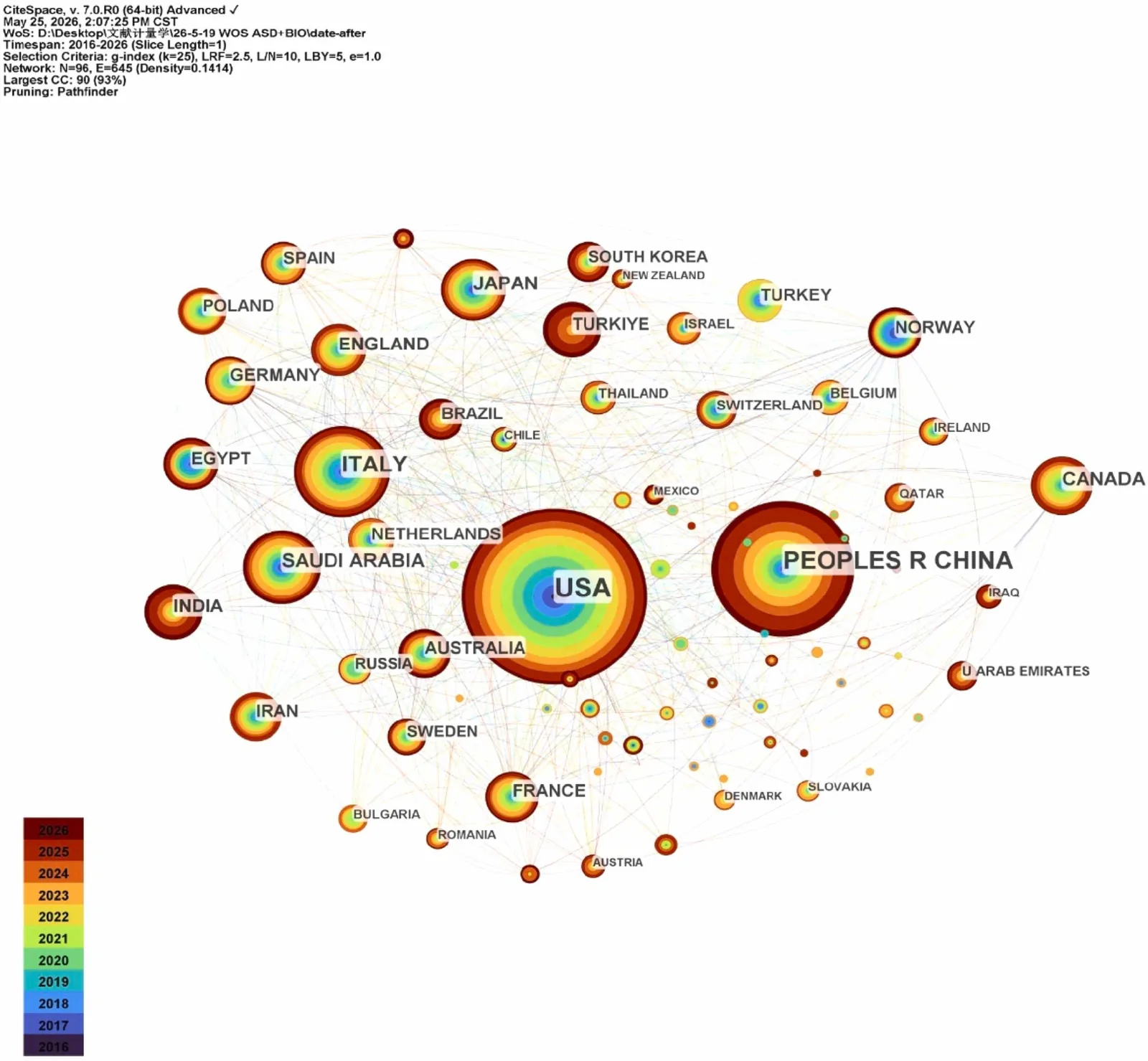
Country/region collaboration network.

Table 1 summarizes the top 10 most productive countries/regions. The United States ranked first with 579 publications and showed the highest centrality value (0.38), indicating its leading role and extensive international collaborations in this field. China ranked second with 348 publications; however, its centrality value was relatively low (0.04), suggesting comparatively weaker connectivity within the global collaboration network. Italy ranked third with 169 publications and a centrality value of 0.15, highlighting its important role in international cooperation. Saudi Arabia and Japan also demonstrated substantial research activity, contributing 99 and 82 publications, respectively. Although India and England produced fewer publications, their relatively high centrality values (0.14 and 0.11, respectively) indicated active participation in international collaborative research.

**Table 1:** Top 10 most productive countries/regions.

Rank	Country	Publications	Centrality
1	USA	579	0.38
Rank	Country	Publications	Centrality
2	China	348	0.04
3	Italy	169	0.15
4	Saudi Arabia	99	0.05
5	Japan	82	0.01
6	Canada	79	0.09
7	India	68	0.14
8	England	62	0.11
9	Germany	59	0.09
10	Egypt	58	0.04

These findings suggest that research on ASD and blood-based biomarkers has developed through extensive international cooperation, with the United States occupying a central position in the global collaboration network.

Nodes represent countries/regions, node size reflects publication output, and connecting lines indicate collaborative relationships between countries/regions.

### Analysis of institutions

The institutional collaboration network related to ASD and blood-based biomarkers is shown in Fig. 4, and the top 10 most productive institutions are summarized in Table 2. The network map demonstrates extensive inter-institutional cooperation, with several major academic centers occupying central positions within the collaboration structure. Node size represents publication output, while the connecting lines indicate collaborative relationships among institutions. The color gradient reflects the temporal distribution of publications, with warmer colors corresponding to more recent research activity.

**Fig. 4:**
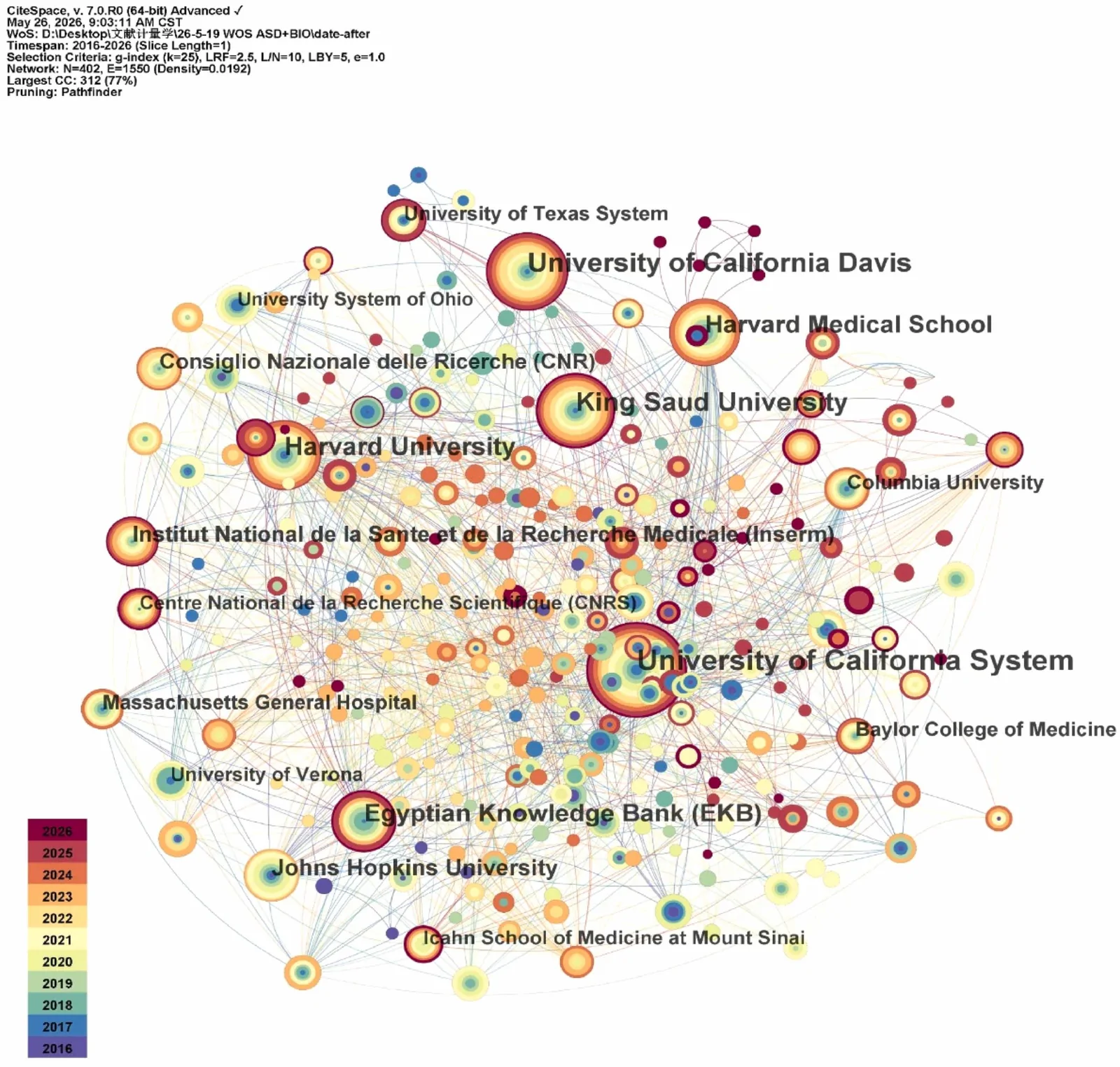
Institutional collaboration network.

**Table 2:** Top 10 most productive institutions.

Rank	Institution	Publications	Centrality
1	University of California System	120	0.1
2	University of California Davis	82	0.02
3	King Saud University	79	0.02
4	Harvard University	65	0.13
5	Egyptian Knowledge Bank (EKB)	56	0.09
6	Harvard University Medical Affiliates	51	0.05
7	Harvard Medical School	50	0.04
Rank	Institution	Publications	Centrality
8	Johns Hopkins University	38	0.09
9	Institut National de la Sante et de la Recherche Medicale (Inserm)	37	0.11
10	Consiglio Nazionale delle Ricerche (CNR)	30	0.07

The University of California System was the most productive institution, contributing 120 publications, and occupied a central position within the network, highlighting its sustained influence in this field. University of California Davis and King Saud University followed with 82 and 79 publications, respectively, indicating substantial research activity and broad collaborative engagement. Harvard University ranked fourth in publication output (65 publications) but exhibited the highest centrality value (0.13), suggesting a stronger role in connecting different research groups and facilitating institutional collaboration.

Although some institutions produced comparatively fewer publications, they demonstrated notable network influence. Institut National de la Santé et de la Recherche Médicale (Inserm) showed a relatively high centrality value (0.11) despite contributing 37 publications, indicating its important bridging function within the collaborative network. Similarly, Egyptian Knowledge Bank (EKB) and Johns Hopkins University both showed centrality values of 0.09, reflecting active participation in international collaborative research. Consiglio Nazionale delle Ricerche (CNR) also maintained moderate connectivity within the network, with a centrality value of 0.07.

The collaboration pattern suggests that ASD and blood-based biomarker research has gradually evolved into a highly interconnected international research field. Institutions with high publication output were not always associated with the highest network centrality, indicating that scientific influence within the collaboration network depends not only on research productivity but also on the extent of institutional cooperation and connectivity. Major universities and research centers, particularly those in the United States and Europe, played important roles in promoting international collaboration and advancing research development in this field.

Nodes represent institutions, node size reflects publication output, and connecting lines indicate collaborative relationships among institutions. Different colors correspond to the year of publication.

### Analysis of journals

Among the co-cited journals in ASD and blood-based biomarkers research, *PLOS ONE* showed the highest co-citation frequency (1109 citations), followed by *Journal of Autism and Developmental Disorders* (954 citations) and *Molecular Psychiatry* (946 citations). Journals with high academic impact, including *Biological Psychiatry*, *Translational Psychiatry*, *Nature*, and *PNAS*, also demonstrated relatively high co-citation frequencies and centrality values, indicating their important positions within the knowledge structure of this field (Table 3).

**Table 3:** Top 10 co-cited journals.

Rank	Journal	Co-citation frequency	Centrality
1	PLOS ONE	1109	0.02
2	J Autism Dev Disord	954	0.01
3	Mol Psychiat	946	0.01
4	Transl Psychiat	830	0.02
5	P NATL ACAD SCI USA	802	0.02
6	Biol Psychiat	796	0.01
7	Nature	774	0.02
8	Autism Res	767	0.01
9	Mol Autism	763	0.01
10	SCI REP-UK	763	0.01

The co-citation network identified *Molecular Psychiatry*, *Biological Psychiatry*, *Brain Research*, *Journal of Neuroscience*, and *Nature Neuroscience* as core journals with substantial network connectivity. In parallel, journals associated with neuroimmune and inflammatory mechanisms, such as *Brain, Behavior, and Immunity*, *Frontiers in Immunology*, and *Journal of Neuroinflammation*, exhibited strong citation associations, reflecting sustained attention toward immune dysregulation, oxidative stress, and neuroinflammatory processes in ASD (Fig. 5A).

**Fig. 5:**
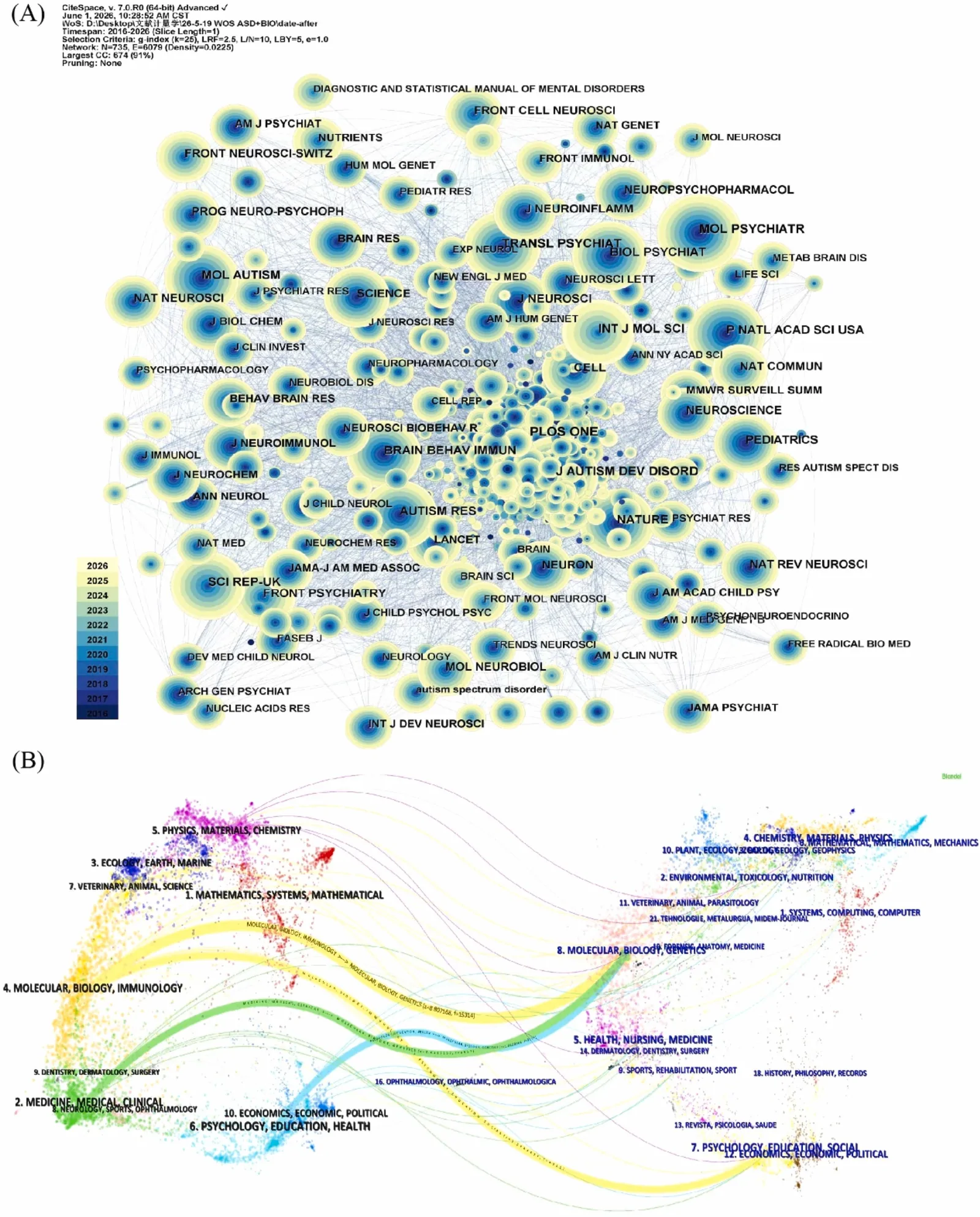
Journal co-citation network and dual-map overlay of journals (A) Nodes represent journals, node size reflects co-citation frequency, and connecting lines indicate co-citation relationships among journals. Different colors correspond to publication years. (B) The left side represents citing journals, and the right side represents cited journals. Colored curves indicate citation pathways among different research disciplines.

The dual-map overlay analysis showed that the major citation trajectories originated from molecular biology, immunology, and neuroscience journals. Over time, these citation pathways gradually extended toward clinical psychiatry and behavioral medicine. Citation links between psychology-related and neuroscience journals were also prominent, indicating increasing integration between neurobiological research and behavioral studies (Fig. 5B).

Collectively, these findings suggest that research on blood-based biomarkers in ASD has progressively evolved from conventional clinical investigations toward mechanistic studies involving molecular pathology, neuroimmune regulation, and translational medicine.

### Analysis of authors and co-cited authors

Author productivity and co-citation patterns were analyzed using VOSviewer (Table 4, Table 5). Among the top 10 authors by the number of publications, Bjorklund G ranks first with 31 articles and is also the most cited author (1470 citations), showing significant academic influence. El-Ansary A, Ashwood P, and Frye RE also show high levels of research output and citation impact (Table 4).

**Table 4:** Top 10 most productive authors.

Rank	Author	Publications	Citations
1	Bjorklund G	31	1470
2	El-ansary A	30	690
3	Ashwood P	28	1291
4	Frye RE	27	1219
5	Bakheet SA	26	691
6	Attia SM	25	677
7	Nadeem A	25	690
8	Ahmad SF	24	500
9	Al-ayadhi L	22	398
10	Al-ayadh LY	21	259

**Table 5:** Top 10 co-cited authors.

Rank	Co-cited author	Co-citations
1	Ashwood P	559
2	Frye R	444
3	Lord C	426
4	Theoharides TC	356
5	Rossignol DA	349
6	El-ansary A	262
7	Bjorklund G	257
8	Jyonouchi H	255
9	Adams JB	253
10	Mostafa GA	252

Co-citation analysis looks at how often two authors are cited together by the same third-party author; the higher the co-citation frequency, the closer their academic interests. The most commonly co-cited author is Ashwood P (559), followed by Frye RE (444) and Lord C (426) (Table 5). Notably, Ashwood P and Frye RE ranked high in both publication count and co-citation frequency, indicating that these two authors have a significant influence in the field.

We built an author collaboration network based on publications with three or more papers (Fig. 6A). Several major collaboration clusters were identified within the network. One cluster centers around Bjorklund G and Bakheet SA, mainly focusing on oxidative stress, inflammation, and metabolic abnormalities in ASD. Another active team, represented by Frye RE, Adams JB, and James SJ, is closely associated with mitochondrial dysfunction and changes in metabolic pathways. Additionally, Ashwood P and Hertz-Picciotto I show strong collaboration in studies involving neuroimmune dysregulation and inflammatory responses. For co-cited authors, we selected those with 30 or more co-citations and created a co-citation author network map (Fig. 6B). As shown, there are four clusters based on color, with the same color indicating close connections. There’s also active collaboration between different co-cited authors, such as Ashwood P, Frye RE, and Lord C.

**Fig. 6:**
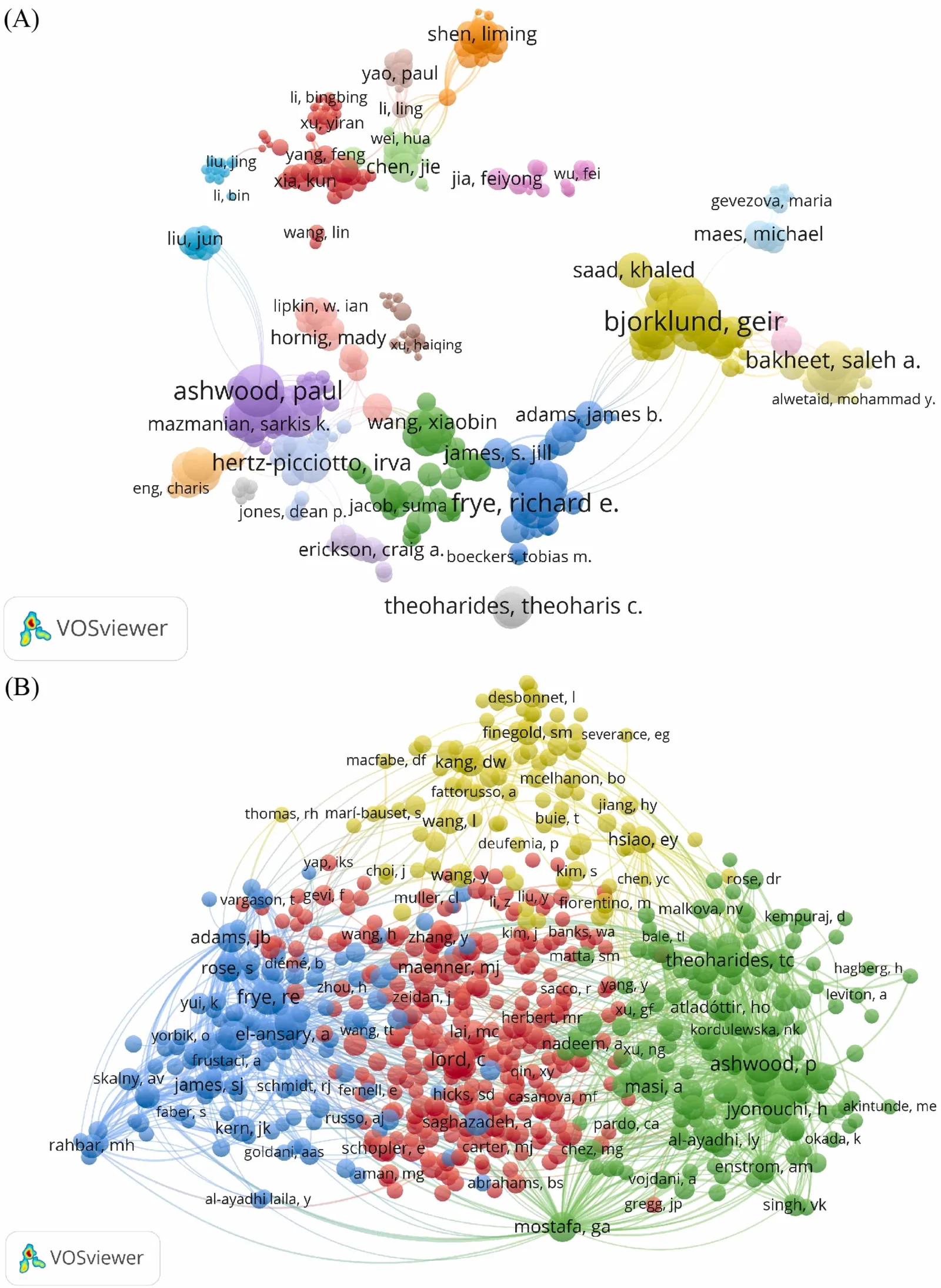
Author collaboration network and Co-cited author network. (A) Nodes represent authors, node size reflects publication output, and connecting lines indicate collaborative relationships among authors. Different colors represent distinct research clusters. (B) Nodes represent co-cited authors, node size reflects co-citation frequency, and connecting lines indicate co-citation relationships among authors. Different colors represent distinct research clusters.

### Analysis of co-cited references and reference clusters

Fig. 7 shows the co-cited reference network and cluster analysis of ASD blood-based biomarker studies. The network included 815 nodes and 3237 links, with a density of 0.0098. The modularity value was 0.653, and the mean silhouette value was 0.7313, indicating a stable clustering structure. Highly co-cited or centrally positioned references included Carter MJ (2014), Wingate M (2014), Baio J (2018), Lord C (2020), Zeidan J (2022), Needham BD (2021), Hughes HK (2023), and Usui N (2023). The node colors showed a time-related distribution, with earlier studies mainly placed in the lower part of the network and more recent studies clustered toward the upper region (Fig. 7A).

**Fig. 7:**
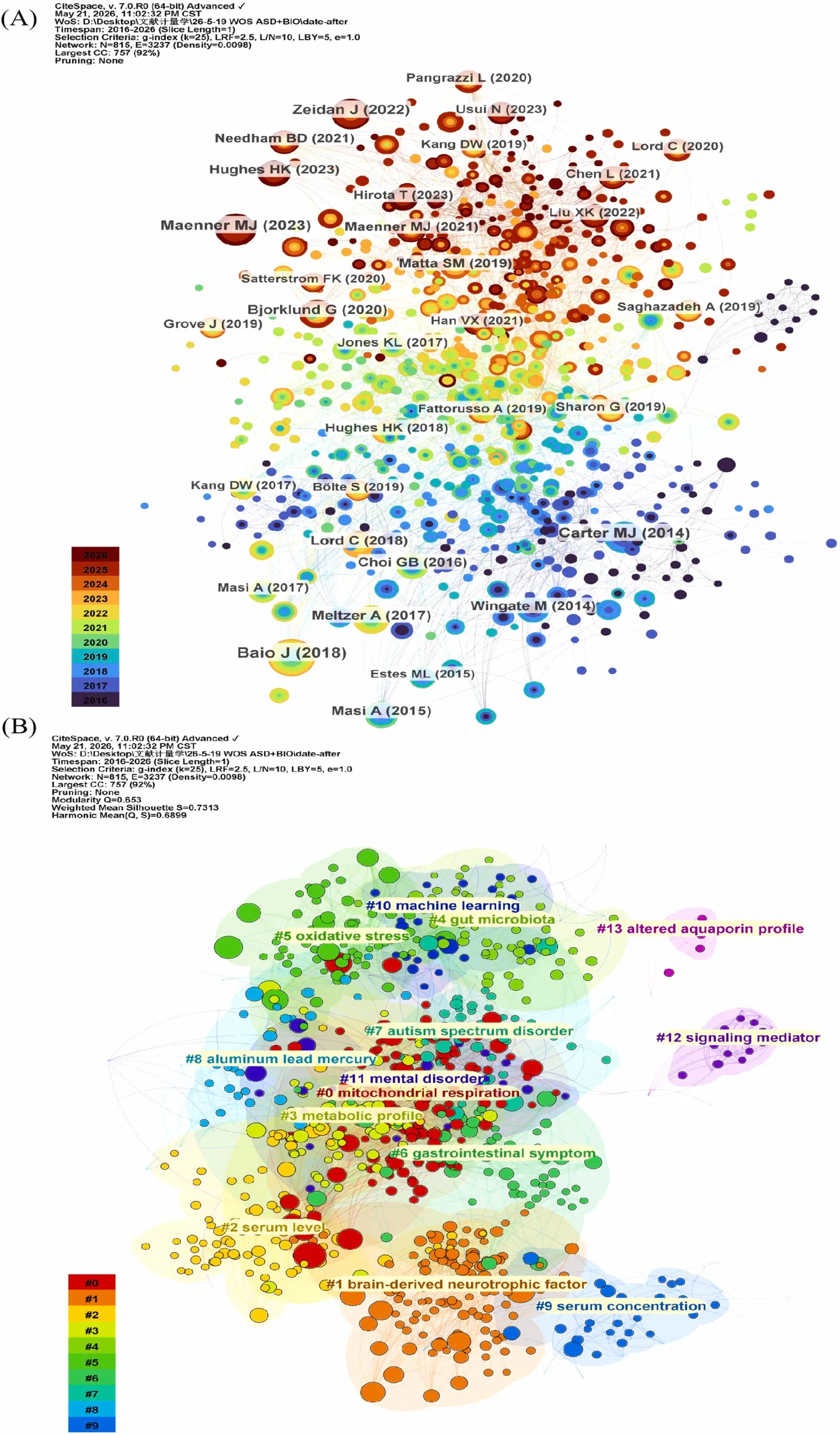
Co-cited reference network and clustering analysis (A) Co-cited reference network of ASD blood-based biomarker studies published from 2016 to 2026. Node size indicates co-citation frequency, and node color represents publication year. (B) Cluster map of co-cited references, showing the main research themes, including mitochondrial respiration, brain-derived neurotrophic factor, serum level, metabolic profile, gut microbiota, oxidative stress, gastrointestinal symptoms, and machine learning.

The co-cited references were grouped into several thematic clusters, mainly involving mitochondrial respiration, brain-derived neurotrophic factor, serum level, oxidative stress, gut microbiota, metabolic profile, gastrointestinal symptoms, machine learning, mental disorder, signaling mediator, and altered aquaporin profile. Central clusters such as oxidative stress, mitochondrial respiration, gut microbiota, metabolic profile, and gastrointestinal symptoms suggest that current ASD biomarker research is closely linked to oxidative damage, immune-metabolic changes, mitochondrial function, and gut–brain interactions (Fig. 7B).

Fig. 8 presents the references with the strongest citation bursts. Earlier bursts were mostly related to ASD epidemiology and foundational research, including Carter MJ (2014), Wingate M (2014) (Wingate et al., 2014), Onore C (2012) (Onore et al., 2012), and Christensen DL (2016) (Christensen et al., 2016). Later bursts increasingly focused on microbiome, metabolism, immune abnormalities, and molecular mechanisms. Recent references with bursts continuing to 2026 included Zeidan J (2022) (Zeidan et al., 2022), Liu XK (2022) (Liu et al., 2022), Hughes HK (2023) (Hughes et al., 2023), Hirota T (2023) (Hirota and King, 2023), Maenner MJ (2023) (Maenner et al., 2023), and Usui N (2023) (Usui et al., 2023). The burst pattern suggests a shift from descriptive and epidemiological studies toward mechanistic and translational research in ASD blood-based biomarkers.

**Fig. 8:**
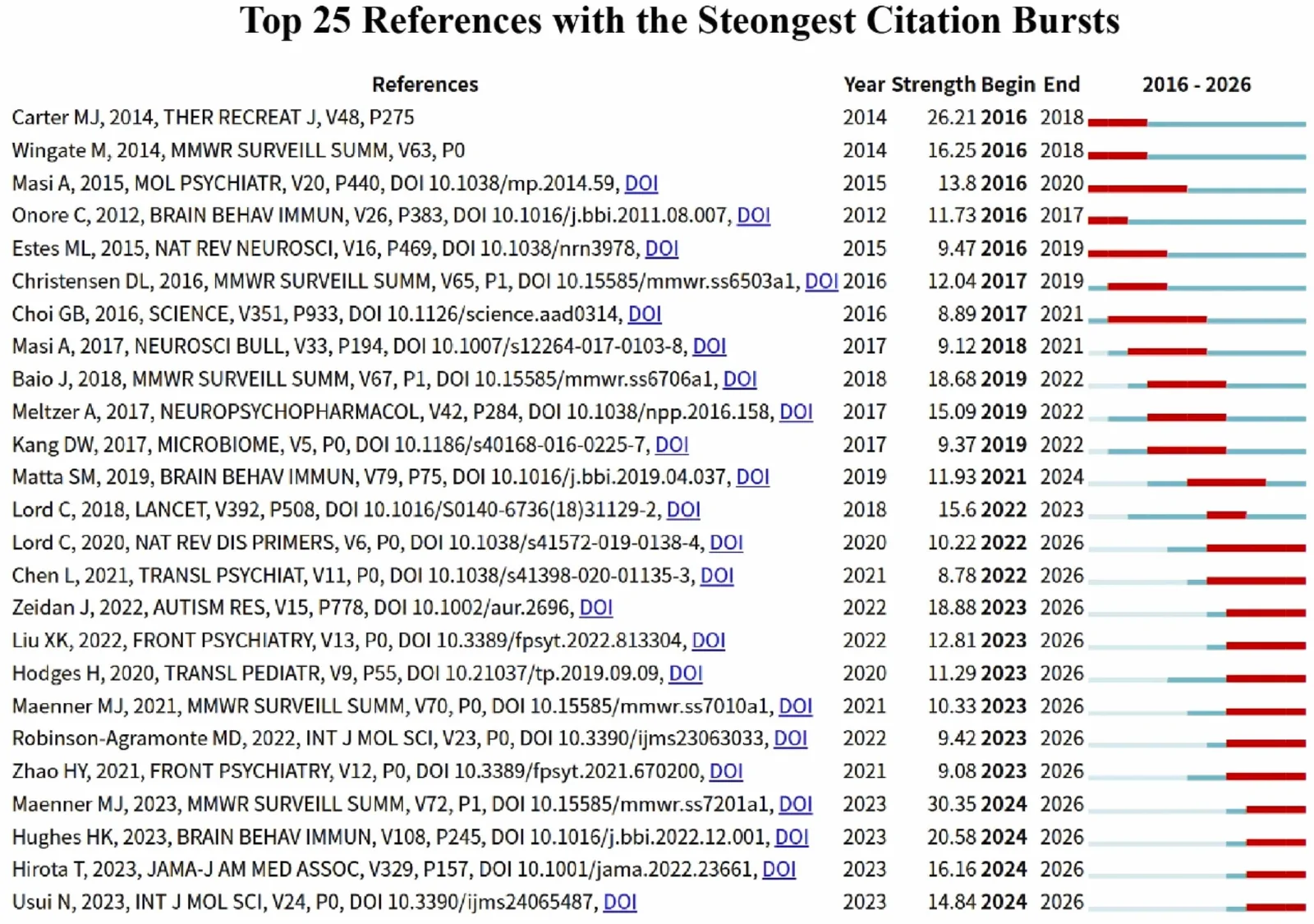
Top 25 references with the strongest citation bursts.

### Analysis of keywords and emerging research trends

The keyword co-occurrence network, clustering analysis, timeline visualization, and keyword burst detection related to ASD and blood-based biomarkers are presented in Fig. 9, Fig. 10.

**Fig. 9:**
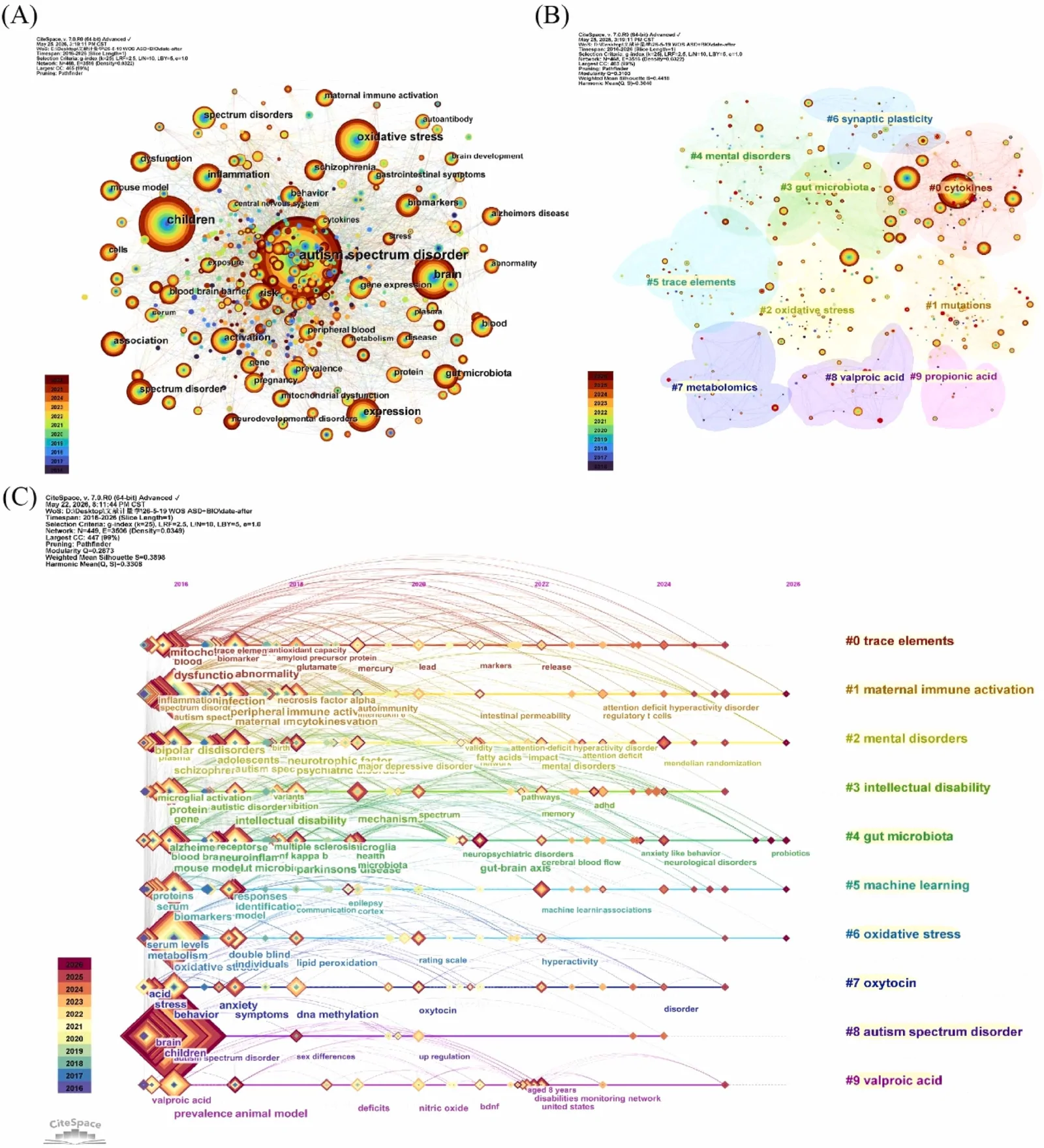
Keyword co-occurrence network, clustering analysis, and timeline visualization (A) Keyword co-occurrence network showing relationships among high-frequency keywords. Node size reflects keyword occurrence frequency, and connecting lines indicate co-occurrence relationships between keywords. Different colors represent publication years. (B) Keyword clustering analysis generated by CiteSpace. Different colors represent distinct research clusters, including cytokines, mutations, oxidative stress, gut microbiota, metabolomics, and synaptic plasticity. (C) Timeline visualization of keyword clusters from 2016 to 2026, illustrating the temporal evolution and development of major research topics in ASD blood-based biomarker studies.

**Fig. 10:**
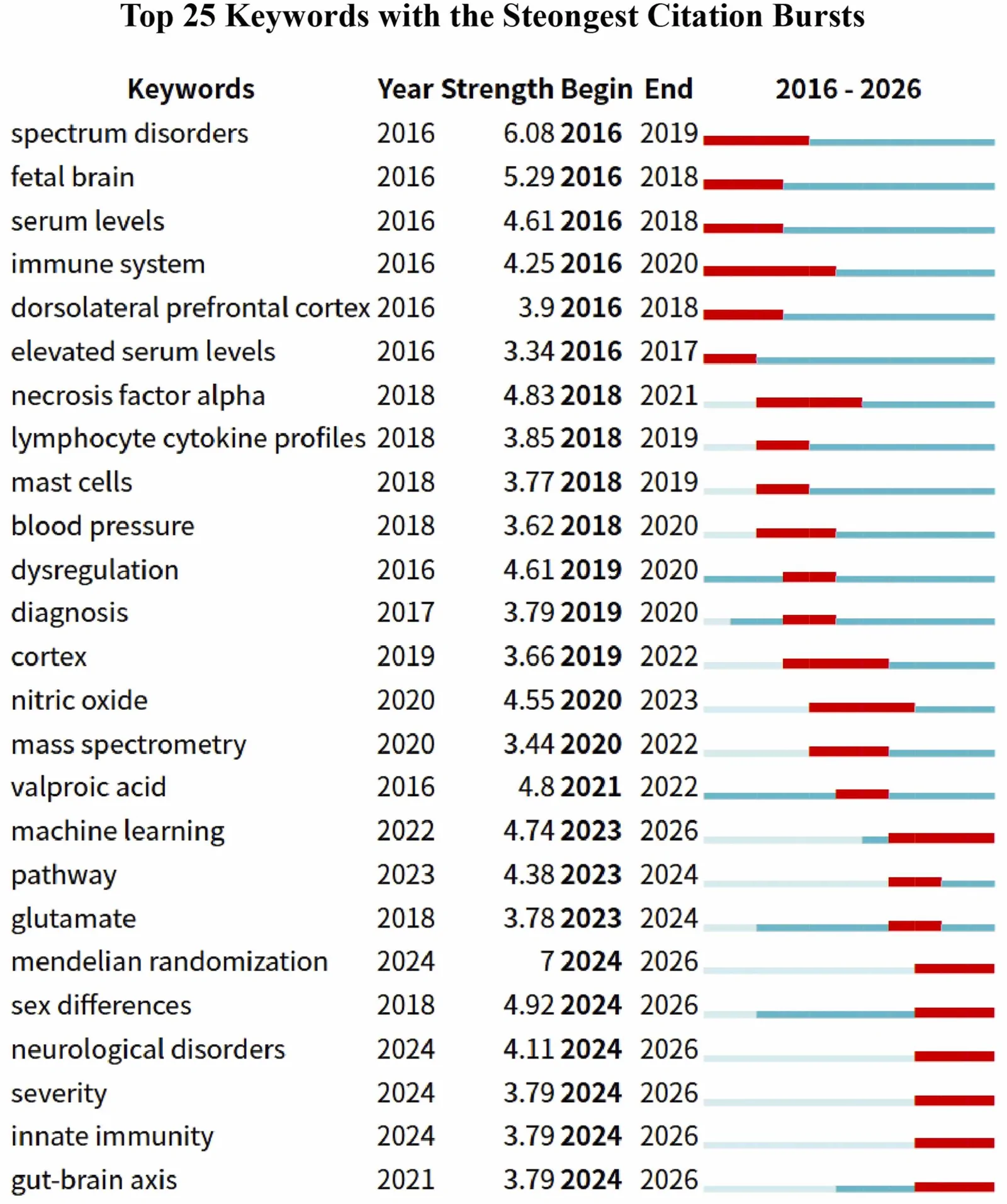
Top 25 keywords with the strongest citation bursts.

High-frequency keywords, including “oxidative stress,” “inflammation,” “children,” “brain,” “gene expression,” “gut microbiota,” and “mitochondrial dysfunction,” occupied central positions within the co-occurrence network, indicating that these topics represent the main research directions in this field. Keyword clustering analysis further identified several major thematic clusters, including “cytokines,” “mutations,” “oxidative stress,” “gut microbiota,” “metabolomics,” “synaptic plasticity,” and “mental disorders.” Among these, clusters related to inflammatory cytokines, oxidative stress, and gut microbiota showed high network density and sustained activity, suggesting continuous attention toward neuroimmune dysregulation and gut–brain interactions in ASD (Fig. 9A, B).

The timeline visualization demonstrated an evident evolution of research topics over time. Earlier studies mainly focused on neurodevelopmental abnormalities, serum biomarkers, immune dysfunction, and diagnostic characteristics. Subsequently, research attention gradually shifted toward mitochondrial dysfunction, oxidative stress, metabolomic alterations, and microbiota-related mechanisms. More recently, mechanistic and translational topics such as “machine learning,” “mendelian randomization,” “gut-brain axis,” “innate immunity,” and “sex differences” have emerged as active research areas (Fig. 9C).

Keyword burst analysis further supported these trends. Early burst keywords included “immune system,” “serum levels,” and “necrosis factor alpha,” whereas recent burst keywords with ongoing activity through 2026 included “machine learning,” “mendelian randomization,” “gut-brain axis,” “innate immunity,” and “neurological disorders.” In particular, “mendelian randomization” showed the highest burst strength (7.0) during the later stage of the study period, indicating growing interest in causal inference and multi-omics approaches in ASD biomarker research (Fig. 10).

After excluding review articles, original studies were stratified into clinical human and preclinical animal-model groups for separate keyword-clustering analyses (Supplementary Figure S1). Clinical studies mainly involved immune and inflammatory responses, machine learning, neuroimmune mechanisms, psychiatric comorbidity, oxidative stress, and gut microbiota-related metabolism. Preclinical studies focused more on maternal immune activation, prenatal exposure, synaptic mechanisms, and genetic and immune mechanisms. Immune-related processes, oxidative stress, and gut microbiota were shared across both groups.

## Discussion

This study analyzed 1785 publications on ASD blood-based biomarkers from 2016 to 2026 using CiteSpace, VOSviewer, and Microsoft Excel. By examining countries, institutions, journals, authors, co-cited references, and keywords, we mapped the global research landscape, highlighted key contributors, and identified emerging areas of interest in this field.

### General research characteristics

The annual output of ASD blood-based biomarker studies showed an overall upward trend from 2016 to 2025, indicating growing interest in biological indicators that may complement behavior-based ASD assessment. This trend is consistent with previous bibliometric studies on ASD-related inflammation and gut microbiota (Chen et al., 2025, Ying et al., 2024), suggesting that biomarker research has become an important part of autism studies.

Country and institutional analyses showed that the United States and China were the two most productive contributors. The United States ranked first in both publication output and centrality, indicating a leading role in international collaboration. China ranked second in publication volume but had lower centrality, suggesting that its global collaborative links remain relatively limited compared with its research output. Major institutions, including the University of California System, Harvard University, Johns Hopkins University, and King Saud University, contributed substantially to this field. The difference between publication output and network centrality also suggests that academic influence depends not only on productivity but also on collaborative connectivity.

Author and co-cited author analyses highlighted several key contributors to ASD blood-based biomarker research. Bjorklund G, El-Ansary A, Ashwood P, Frye RE, and Bakheet SA were among the most productive authors, whereas Ashwood P, Frye RE, Lord C, Theoharides TC, Rossignol DA, and El-Ansary A were frequently co-cited. Their work mainly focused on oxidative stress, immune dysregulation, mitochondrial dysfunction, metabolic abnormalities, and ASD clinical phenotyping. Rossignol DA and Frye RE contributed to the understanding of mitochondrial dysfunction in ASD, suggesting that impaired cellular energy metabolism may be involved in ASD pathophysiology (Frye et al., 2024a). Evidence from Masi A and Vargas DL further linked ASD with inflammatory cytokine abnormalities and glial activation, providing a basis for later biomarker studies on neuroimmune mechanisms (Masi et al., 2015, Vargas and Nascimbene, 2005). These findings indicate that the field has been driven by several stable research groups, but broader collaboration is still needed to improve data sharing and reproducibility.

Journal analysis also showed the interdisciplinary nature of ASD blood-based biomarker research. Highly co-cited journals included *PLOS ONE, Journal of Autism and Developmental Disorders, Molecular Psychiatry, Translational Psychiatry, Biological Psychiatry,* and *Nature*. These journals cover molecular biology, psychiatry, neuroscience, immunology, and clinical medicine. The dual-map overlay suggested that the knowledge base of this field mainly comes from molecular biology, immunology, and neuroscience, and has gradually extended toward clinical psychiatry and behavioral medicine. This pattern supports the view that ASD is a biologically heterogeneous neurodevelopmental condition involving multiple interacting systems.

### Core research hotspots

Co-cited reference and keyword analyses showed that oxidative stress, mitochondrial dysfunction, inflammatory responses, gut microbiota, metabolic profiles, and gastrointestinal symptoms were the main knowledge clusters. These connected themes suggest that ASD blood-based biomarker research is shifting from descriptive clinical features toward biological mechanisms underlying atypical neurodevelopment.

Oxidative stress emerged as one of the most stable and highly connected themes throughout the study period. Keywords including “oxidative stress,” “glutathione,” “redox imbalance,” and “lipid peroxidation” appeared frequently in keyword co-occurrence analyses. Previous studies have reported impaired antioxidant defense capacity and abnormal redox metabolism in individuals with ASD (Mandic-Maravic et al., 2021). Frye and Wang J suggested that oxidative imbalance may interfere with neuronal signaling, synaptic plasticity, and neurodevelopmental regulation (Frye et al., 2024a, Wang et al., 2022). In parallel, Masi et al. and Takada observed elevated inflammatory cytokines together with oxidative abnormalities in ASD populations, indicating potential interactions between immune activation and oxidative stress pathways (Masi et al., 2015, Takada et al., 2024). Despite these findings, oxidative stress markers still lack sufficient disease specificity because comparable abnormalities have also been detected in schizophrenia, bipolar disorder, and attention-deficit/hyperactivity disorder (Munkholm et al., 2024, Palaniyappan, 2022, Suwindi et al., 2026). Although oxidative stress appears to represent an important biological component in ASD pathophysiology, current evidence remains insufficient to support its use as an independent diagnostic biomarker because of limited specificity and substantial inter-study heterogeneity (Parellada et al., 2023, Rossignol and Frye, 2014). Their potential value may instead lie in identifying biologically distinct ASD subgroups and monitoring therapeutic responses.

Mitochondrial dysfunction represented another major hotspot in the present analysis. Keywords related to “mitochondrial dysfunction” and “energy metabolism” showed high occurrence frequency together with strong citation bursts. Earlier studies suggested that mitochondrial abnormalities may underlie developmental regression, fatigue, gastrointestinal symptoms, and atypical neurodevelopmental trajectories in some children with ASD (Khaliulin et al., 2025). Multiple investigations have reported abnormalities in mitochondrial-related metabolic markers, including pyruvate, carnitine, and coenzyme Q10, among ASD populations (Aldred et al., 2003). Correia et al. detected elevated plasma lactate levels and increased lactate-to-pyruvate ratios in a subset of children with ASD, suggesting impaired aerobic respiration and altered cellular energy metabolism (Correia et al., 2006). Muscle biopsy analyses further identified mitochondrial defects in several autistic children (Tsao and Mendell, 2007). Subsequent studies reported biochemical evidence of impaired oxidative phosphorylation in a proportion of ASD individuals (Haas, 2010). In addition, alterations in mitochondrial DNA copy number, mtDNA deletions, and other mitochondrial genetic abnormalities have been reported in subsets of individuals with ASD (Deneubourg et al., 2022, Gevezova et al., 2020, Viering et al., 2023). Collectively, these findings indicate that disrupted cellular energy metabolism may influence neuronal maturation and synaptic development during critical neurodevelopmental stages.

The increasing prominence of metabolomics-related keywords also indicates that ASD biomarker research is gradually shifting from isolated marker exploration toward systems biology approaches. Previous studies have demonstrated that individuals with ASD exhibit distinct abnormalities in amino acid metabolism, which may contribute to alterations in neurological function and behavioral outcomes (Chang et al., 2024, Frye and Rose, 2024, Prince et al., 2023). These metabolic disturbances are mainly reflected by dysregulation of specific amino acid pathways, including increased, decreased, or disrupted metabolic activity. For instance, elevated concentrations of phenylalanine and glutamate have been observed in some children with ASD (Nisar et al., 2022). Altered tryptophan metabolism has also received increasing attention in recent years. Several studies reported reduced tryptophan levels in peripheral blood and cerebrospinal fluid in children with ASD, which may subsequently affect serotonin synthesis and metabolic regulation (Kaluzna-Czaplinska et al., 2017, Muller et al., 2016). Because serotonin plays an important role in social communication, emotional regulation, and neurodevelopment, disturbances in this pathway may partly explain several core behavioral features observed in ASD (Al-Beltagi et al., 2024).

The gut–brain axis has become another rapidly developing research direction in recent years. Keywords related to “gut microbiota,” “short-chain fatty acids,” “microbial metabolites,” and “gastrointestinal symptoms” increased substantially during the later study period. Highly cited studies by Hsiao et al., Sharon et al., and Kang et al. demonstrated that alterations in gut microbial composition may influence ASD-like behaviors and gastrointestinal symptoms (Hsiao et al., 2013, Kang et al., 2017, Sharon and Cruz, 2019). These findings support the gut–brain axis hypothesis, which proposes that intestinal microbiota may regulate brain development through metabolic, immune, and neural signaling pathways (Wang et al., 2023).

Despite increasing evidence, the causal relationship between gut microbiota abnormalities and ASD remains controversial. Dietary selectivity, chronic constipation, antibiotic exposure, and lifestyle differences may substantially influence microbial composition. Consequently, current evidence mainly supports an association between ASD and gut microbial dysbiosis rather than establishing a direct causal relationship.

To further assess the consistency of evidence within the major biological domains highlighted above, we qualitatively examined representative influential references for oxidative stress, inflammatory cytokines, mitochondrial dysfunction, and gut microbiota (Supplementary Table 1). Oxidative stress findings are relatively consistent at the pathway level, particularly for glutathione-related redox imbalance, although results for individual markers remain variable (Chen et al., 2021, Frustaci et al., 2012, Rossignol and Frye, 2012b). Findings for inflammatory cytokines are less consistent, with differences across cytokines, sample types, and clinical subgroups (Jones et al., 2017, Masi et al., 2015, Saghazadeh et al., 2019). Mitochondrial abnormalities have been repeatedly reported, but they appear to occur mainly in specific subsets of individuals with ASD rather than across the spectrum as a whole (Frye, 2020, Frye et al., 2024a, Rossignol and Frye, 2012a). Gut microbiota studies generally support an association between ASD and microbial dysbiosis, yet the specific microbial changes reported across studies remain inconsistent and are influenced by both clinical and methodological factors (Fattorusso et al., 2019, Kang et al., 2017, Sharon and Cruz, 2019). Overall, these findings support the biological relevance of the four research domains, while also showing that the strength and consistency of evidence vary considerably among them.

The stratified analysis provides additional context for interpreting these bibliometric patterns. Although clinical and preclinical studies shared several biological themes, their research emphasis differed. Clinical studies tended to focus more on measurable peripheral biomarkers, clinical phenotypes, and emerging prediction approaches, whereas preclinical work was more often directed toward developmental exposures and mechanistic pathways. This distinction is relevant to clinical translation. Animal models can help clarify biological mechanisms and identify candidate pathways, but preclinical findings do not necessarily translate directly into diagnostic or prognostic utility in humans (Leenaars et al., 2019). Their clinical relevance therefore requires further validation in well-characterized human cohorts.

### Biomarker specificity and potential clinical utility

Although oxidative stress, mitochondrial dysfunction, inflammatory responses, and gut–brain interactions were prominent themes in our analysis, these abnormalities are not unique to ASD. Similar redox abnormalities have been reported in schizophrenia and bipolar disorder (Das et al., 2019, Jimenez-Fernandez et al., 2021), while mitochondrial and lactate abnormalities have also been described in these disorders (Dogan et al., 2018, Kuang et al., 2018). Gut microbial dysbiosis is likewise not confined to ASD, as altered gut microbial composition has also been reported in psychosis and schizophrenia (Murray et al., 2023), and inflammatory abnormalities overlap with other neurodevelopmental disorders (Gagliano et al., 2025). These findings suggest that the major clusters identified here may reflect broader neurobiological dysfunction rather than ASD-specific diagnostic signatures.

Limited disease specificity, however, does not exclude clinical value. Mitochondrial biomarkers may be useful for identifying biologically distinct subgroups, including those characterized by neurodevelopmental regression or abnormal acyl-carnitine profiles (Frye et al., 2024a). Inflammatory markers may help characterize immune-related phenotypes, although their relationship with autism severity remains inconsistent (Saghazadeh et al., 2019, Zhao et al., 2021). Gut microbiota may be more useful for phenotype stratification than diagnosis because ASD-associated microbial signatures vary with age, sex, and bowel function (West et al., 2022). Oxidative stress markers have also shown preliminary potential for treatment monitoring (Bertoglio et al., 2010). More broadly, a systematic review identified 940 candidate response biomarkers across 280 ASD studies, 755 of which were reported in only one publication (Parellada et al., 2023). These findings suggest that blood-based biomarkers may have greater near-term value for subgroup identification, treatment-response assessment, and longitudinal monitoring than for stand-alone diagnosis.

The limited specificity of individual biomarkers also supports the development of multimarker models. Recent plasma proteomic and metabolomic studies have used machine-learning approaches to combine multiple biological signals and reported promising classification performance (Liu et al., 2024, Tang et al., 2025). Compared with single-marker approaches, multimarker models may improve the ability to distinguish ASD-related biological patterns and may also enhance their potential for clinical translation by better capturing the heterogeneity of ASD. This need to integrate multiple, partially informative biomarkers also provides a rationale for the growing use of machine-learning approaches in ASD biomarker research.

### Machine learning and biomarker prediction models

Citation burst and keyword burst analyses indicate a shift from epidemiology, diagnosis, and clinical description toward molecular mechanisms and translational applications. Earlier burst references were mainly related to ASD prevalence, diagnostic frameworks, and foundational clinical studies. More recent burst keywords involved microbiome, metabolism, immune abnormalities, molecular pathways, machine learning, and Mendelian randomization.

Machine learning has become one of the most active emerging topics. Keywords such as “machine learning,” “prediction model,” and “precision medicine” demonstrated strong citation bursts in recent years, suggesting growing interest in data-driven approaches within ASD biomarker research.

Traditional biomarker studies often focus on isolated biological indicators with limited diagnostic sensitivity and specificity. Machine learning approaches enable researchers to integrate multidimensional datasets across different biological domains. These datasets may include inflammatory cytokines, metabolomic and transcriptomic profiles, gut microbiota-derived metabolites, neuroimaging findings, and behavioral characteristics (Reel et al., 2021). Recent studies have suggested that algorithms such as support vector machines, random forest models, and neural networks may improve ASD classification accuracy and facilitate biological subtype identification (Xiong et al., 2025, Zhang et al., 2024). However, current machine learning studies still face several limitations. Many studies rely on relatively small sample sizes and lack independent external validation cohorts (Shirwaikar et al., 2023). Differences in preprocessing methods and feature selection strategies may further reduce reproducibility. Therefore, future computational biomarker studies should prioritize standardized analytical pipelines, transparent reporting standards, and large multicenter collaborations before clinical implementation can be considered.

The bibliometric patterns observed in this study, together with the qualitative evidence discussed above, suggest that these research domains are at different stages of development and validation. Long-standing themes such as oxidative stress and mitochondrial dysfunction showed sustained prominence in the co-citation and keyword analyses and have accumulated relatively broad evidence bases, although their limited disease specificity and between-study variability continue to restrict clinical translation. In contrast, microbiome-based biomarkers, multimarker models, and particularly machine-learning approaches have gained more recent attention and remain at an earlier stage of validation. Their growing bibliometric prominence should therefore be interpreted as increasing research interest rather than evidence of clinical readiness.

### Future research directions

Several priorities for future research arise directly from the bibliometric patterns observed in this study. Research activity was concentrated in several leading countries, institutions, and author groups, while international collaboration remained uneven. This concentration highlights the need for replication across independent, multicenter, and geographically diverse cohorts to determine whether biomarker findings are reproducible across populations and research settings. Beyond improving reproducibility, future studies should focus more directly on clinical utility. The value of blood-based biomarkers depends on whether they can support early screening, biological subgrouping, individualized intervention, treatment-response prediction, or long-term outcome evaluation. At present, no blood-based biomarker is ready for routine clinical use in ASD. Standardized laboratory procedures, transparent computational workflows, and external validation are still needed before clinical translation can be achieved.

### Strengths and limitations

This study offers a broad overview of research on blood-based biomarkers in ASD over the past decade using co-citation analysis, keyword clustering, collaboration mapping, and burst detection. Several limitations should be kept in mind when interpreting the findings. The analysis was limited to English-language publications indexed in the Web of Science Core Collection, so relevant studies from other databases or languages may have been missed. The dataset also included both original articles and reviews, with reviews accounting for about one fifth of the included literature. Including reviews helped provide a more complete picture of the field, but it may also have affected citation-based analyses because reviews tend to synthesize and cite a broader range of previous work. Prominent clusters should therefore be viewed mainly as areas that have attracted sustained research attention, rather than as direct evidence that the underlying findings are stronger or more consistent. Citation-based indicators may also be shaped by publication age, citation practices, and journal visibility. More importantly, bibliometric analysis can map research activity and knowledge structure, but it cannot by itself establish the methodological quality, reproducibility, or clinical usefulness of individual biomarkers.

## Conclusions

This bibliometric analysis outlined the development of ASD blood-based biomarker research from 2016 to 2026. Publication output increased steadily during this period, with the United States and China contributing the most studies. The United States also held a more central position in international collaboration, while participation from other countries and institutions continued to expand.

The main knowledge base of the field centered on oxidative stress, mitochondrial dysfunction, inflammatory responses, gut microbiota, metabolic profiles, and gastrointestinal symptoms. These findings suggest that ASD biomarker research has moved beyond the search for isolated blood markers. Current studies pay more attention to immune-metabolic regulation, mitochondrial function, gut–brain interactions, and the biological heterogeneity of ASD.

Recent trends show a further shift toward mechanistic and translational research. Machine learning, Mendelian randomization, innate immunity, sex differences, and the gut–brain axis have become active topics. These directions reflect growing interest in causal inference, multi-omics integration, and data-driven biomarker prediction, which may help identify more reliable biomarker panels and biologically meaningful ASD subgroups.

At present, blood-based biomarkers are not yet ready for routine clinical use in ASD. The field is still limited by heterogeneous study populations, small sample sizes, inconsistent analytical methods, and insufficient external validation. Future studies should place greater emphasis on multicenter longitudinal cohorts, standardized experimental and computational workflows, detailed clinical phenotyping, and independent validation. Such work will be important for improving reproducibility and moving ASD blood-based biomarkers closer to clinical application.

## Author contributions

LX and JYZ: conceptualization. HBH and YYH: data curation and formal analysis. YYC and YW: investigation and methodology. RW, HZ, and BQY: software and visualization. HTL and HJ: supervision and project administration. LX: writing—original draft. JYZ and HBH: data interpretation and writing—review and editing. All authors read and approved the final manuscript. LX, JYZ, and HBH contributed equally to this work as co-first authors.

## CRediT authorship contribution statement

**Lei Xu:** Writing – original draft, Conceptualization. **Jia-Ying Zhang:** Writing – review & editing, Data curation, Conceptualization. **Hong-Biao Huang:** Writing – review & editing, Formal analysis, Data curation. **Yuan-Yuan Huang:** Formal analysis, Data curation. **Yi-Yuan Chen:** Methodology, Investigation. **Ying Wu:** Methodology, Investigation. **Ran Wei:** Visualization, Software. **Hui Zhong:** Visualization, Software. **Bo-Qiu Yan:** Visualization, Software. **Hai-Tao Lv:** Supervision, Project administration. **Hua Jin:** Supervision, Project administration.

## Funding

This project was funded by the Applied Foundational Research of Medical and Health Care of Suzhou City (SYW2025051).

## Declaration of Competing Interest

The authors declare no competing interests.

## Footnotes

Appendix A Supplementary data associated with this article can be found in the online version at doi:10.1016/j.ibneur.2026.09.012.
