# Supplementary material for: Mapping the knowledge structure and emerging trends of blood-based biomarkers in autism spectrum disorder: A bibliometric analysis (2016–2026)
Source: IBRO Neurosci Rep. 2026 Sep 24;21:884–900. doi: 10.1016/j.ibneur.2026.09.012 (PMC13639166; doi:10.1016/j.ibneur.2026.09.012)
Supplement: Supplementary file 1 [file mmc1.docx]

Supplementary Table1. Representative influential references and qualitative interpretation of major biological knowledge clusters in ASD blood-based biomarker research

| Knowledge cluster | Representative influential references | Overall pattern | Qualitative interpretation |
| --- | --- | --- | --- |
| Oxidative stress | Chen L et al., 2021; Rossignol DA & Frye RE, 2012; Frustaci A et al., 2012(Chen L. et al., 2021;Frustaci A. et al., 2012;Rossignol D. A. and Frye R. E., 2012b) | Moderately consistent | Glutathione-related redox abnormalities are repeatedly reported, but individual oxidative markers remain heterogeneous. The evidence supports redox imbalance as an important biological feature rather than an ASD-specific marker. |
| Inflammatory cytokines | Masi A et al., 2015; Jones KL et al., 2017; Saghazadeh A et al., 2019(Jones K. L. et al., 2017;Masi A. et al., 2015;Saghazadeh A. et al., 2019) | Partially consistent | Several cytokines show group-level alterations, but findings vary by cytokine, sample type, and clinical subgroup. Current evidence supports immune dysregulation, but not a uniform ASD-specific cytokine profile. |
| Mitochondrial respiration / dysfunction | Frye RE, 2020; Rossignol DA & Frye RE, 2012; Frye RE et al., 2024(Frye R. E., 2020;Frye R. E. et al., 2024a;Rossignol D. A. and Frye R. E., 2012a) | Consistent mainly at subgroup level | Mitochondrial and bioenergetic abnormalities are repeatedly reported in subsets of ASD, but their type and prevalence vary. The evidence favors distinct mitochondrial phenotypes rather than a uniform defect across ASD. |
| Gut microbiota | Sharon G et al., 2019; Fattorusso A et al., 2019; Kang DW et al., 2017(Fattorusso A. et al., 2019;Kang D. W. et al., 2017;Sharon G. et al., 2019) | Association relatively consistent; specific signatures inconsistent | Studies generally support gut microbial dysbiosis in ASD, but specific taxa are poorly replicated and strongly influenced by clinical and methodological factors. The gut–brain axis is biologically relevant, but no stable ASD-specific microbial signature has been established. |

Note: Representative references, including original studies, reviews, and meta-analyses, were selected from the co-citation dataset with consideration of co-citation frequency, citation burst strength, centrality, and thematic relevance.

Supplementary Figure S1

Supplementary Figure S1. Keyword clustering analysis of clinical and preclinical studies generated by CiteSpace. Different colors represent distinct research clusters. (A) Clinical studies. (B) Preclinical animal-model studies.
